# Optimizing shoulder elevation assist rate in exoskeletal rehabilitation based on muscular activity indices: a clinical feasibility study

**DOI:** 10.1186/s12883-024-03651-x

**Published:** 2024-05-09

**Authors:** Daisuke Ito, Mori Fukuda, Yuichiro Hosoi, Ryotaro Hirose, Tatsuya Teramae, Takayuki Kamimoto, Yuka Yamada, Tetsuya Tsuji, Tomoyuki Noda, Michiyuki Kawakami

**Affiliations:** 1 Department of Rehabilitation Medicine, Keio University School of Medicine, 35 Shinanomachi, Shinjuku-ku, Tokyo, 160-8582 Japan; 2 Graduate School of Science and Technology, Keio University, Tokyo, Kanagawa Japan; 3 Department of Brain Robot Interface, Brain Information Communication Research Laboratory Group, Advanced Telecommunications Research Institute International, Kyoto, Japan

**Keywords:** Stroke, Shoulder, Robotics, Rehabilitation, Feasibility studies

## Abstract

**Background:**

Restoring shoulder function is critical for upper-extremity rehabilitation following a stroke. The complex musculoskeletal anatomy of the shoulder presents a challenge for safely assisting elevation movements through robotic interventions. The level of shoulder elevation assistance in rehabilitation is often based on clinical judgment. There is no standardized method for deriving an optimal level of assistance, underscoring the importance of addressing abnormal movements during shoulder elevation, such as abnormal synergies and compensatory actions. This study aimed to investigate the effectiveness and safety of a newly developed shoulder elevation exoskeleton robot by applying a novel optimization technique derived from the muscle synergy index.

**Methods:**

Twelve chronic stroke participants underwent an intervention consisting of 100 robot-assisted shoulder elevation exercises (10 × 10 times, approximately 40 min) for 10 days (4–5 times/week). The optimal robot assist rate was derived by detecting the change points using the co-contraction index, calculated from electromyogram (EMG) data obtained from the anterior deltoid and biceps brachii muscles during shoulder elevation at the initial evaluation. The primary outcomes were the Fugl–Meyer assessment-upper extremity (FMA-UE) shoulder/elbow/forearm score, kinematic outcomes (maximum angle of voluntary shoulder flexion and elbow flexion ratio during shoulder elevation), and shoulder pain outcomes (pain-free passive shoulder flexion range of motion [ROM] and visual analogue scale for pain severity during shoulder flexion). The effectiveness and safety of robotic therapy were examined using the Wilcoxon signed-rank sum test.

**Results:**

All 12 patients completed the procedure without any adverse events. Two participants were excluded from the analysis because the EMG of the biceps brachii was not obtained. Ten participants (five men and five women; mean age: 57.0 [5.5] years; mean FMA-UE total score: 18.7 [10.5] points) showed significant improvement in the FMA-UE shoulder/elbow/forearm score, kinematic outcomes, and pain-free passive shoulder flexion ROM (*P* < 0.05). The shoulder pain outcomes remained unchanged or improved in all patients.

**Conclusions:**

The study presents a method for deriving the optimal robotic assist rate. Rehabilitation using a shoulder robot based on this derived optimal assist rate showed the possibility of safely improving the upper-extremity function in patients with severe stroke in the chronic phase.

**Supplementary Information:**

The online version contains supplementary material available at 10.1186/s12883-024-03651-x.

## Background

Stroke is a leading cause of disability [1, 2], and approximately 70% of patients with stroke experience upper-extremity dysfunction as their primary symptom [3, 4]. This dysfunction significantly impacts their activities of daily living [5] and overall health-related quality of life [6]. Moreover, patients have reported that losing upper-extremity function is one of the most distressing long-term consequences [7]. Therefore, improving upper-extremity function has become a critical concern for both stroke survivors and their caregivers.

Robotic rehabilitation has become an effective functional training method for improving upper-extremity function. The American Heart Association Guideline 2016 indicates that robotic therapy is reasonable for delivering more upper-extremity intensive practice in patients with stroke with moderate to severe paresis [8]. A previous study reported that higher-intensity robotic therapy exercises improved upper-extremity impairment after stroke [9]. However, the effectiveness of robotic therapy has focused on the distal hand and fingers, and no significant efficacy of robotic therapy for hemiplegic shoulders has been elucidated [10]. In particular, it is difficult to safely assist shoulder elevation against gravity (vertical movement) through robotic intervention because of the complex musculoskeletal anatomy of the shoulder. Functional upper-extremity activity requires control of the proximal segments to position and orient the hand to the environment [11]. Moreover, distal hand function depends on proximal shoulder function [12]. Therefore, establishing a robotic rehabilitation strategy to improve shoulder elevation movements in patients with stroke is essential.

The difficulty of developing shoulder robots lies in determining the optimal assistance rate. The level of shoulder elevation assistance in rehabilitation is often based on clinical judgment, and there is no standardized method for deriving the optimal level of assistance. Recently, robots equipped with the assist-as-needed algorithm have been developed [13, 14]. However, none of these have determined a quantitative robot assist rate. Hence, we focused on addressing abnormal movements, such as abnormal synergies and compensatory actions during shoulder elevation. Abnormal synergies are attempts at individual movements of one body part accompanied by excessive, unintended motions of contiguous body parts [15, 16]. In stroke survivors, abnormal synergies are commonly described between the shoulder and elbow muscles [17], which are the leading causes of reaching dysfunctions [18, 19].

Moreover, compensatory actions, such as elbow flexion, are frequently observed during hemiplegic shoulder flexion [20], and the excessive use of compensatory actions leads to a reduced range of joint motion, pain, and limited functional improvement in the hemiplegic upper-extremity [21]. Therefore, abnormal movements during shoulder elevation should be minimized. It is feasible to increase robotic assistance to reduce abnormal movements. Robotic rehabilitation for severe upper-extremity paralysis has been improved with an error-reduction strategy (adding more robotic assistance and minimizing errors) [22]. Conversely, it has been demonstrated that excessive robot assistance during intentional movements may lead to “slacking,” a condition of reduced motor output, energy expenditure, and own participation [23, 24]. Therefore, optimal robotic assistance improves upper-extremity function after stroke [25], particularly in hemiplegic shoulder elevation movements. However, no established methods exist for determining the robot assist rate based on abnormal movements during shoulder elevation. This study aimed to investigate the effectiveness and safety of an intervention using a newly developed shoulder elevation-assisted exoskeletal robot by applying a novel optimization technique derived from the muscle synergy index.

## Methods

### Study design and participants

This study used a single-group pre-test and post-test feasibility design. The inclusion criteria were as follows: (1) age 20–80 years; (2) patients with hemiplegia because of the unilateral hemispheric lesion; (3) some mobility in the upper-extremity (Stroke Impairment Assessment Set motor score of proximal upper-extremity ≥ 2), (4) passive range of motion (ROM) of shoulder flexion ≥ 120º, (5) understand the purpose of the study (no severe aphasia or cognitive impairment). The exclusion criteria were obvious shoulder pain or inflammation, a history of orthopedic diseases such as shoulder luxation, fracture, or traumatic rotator cuff injury, contraindications to exercise therapy and disturbance of consciousness. This study was conducted according to the Declaration of Helsinki and was reviewed and approved by the Ethics Committee of Keio University (Approval Number: 20,180,360). All participants provided written informed consent before data collection and intervention.

### Experimental protocol

The experiment consisted of pre-evaluation, ten days of shoulder elevation-assisted exoskeletal robotic intervention, and post-evaluation for 12 days. The intervention frequency of the sessions was four to five times per week, and the experiment was completed within three weeks. Moreover, concomitant therapy, including traditional occupational therapy and functional electrical stimulation of the extensor digitorum muscle, was performed five times a week for 60 min, in addition to robotic rehabilitation.

### Intervention protocol

#### Shoulder elevation-assisted exoskeletal Robot

**Fig. 1:**
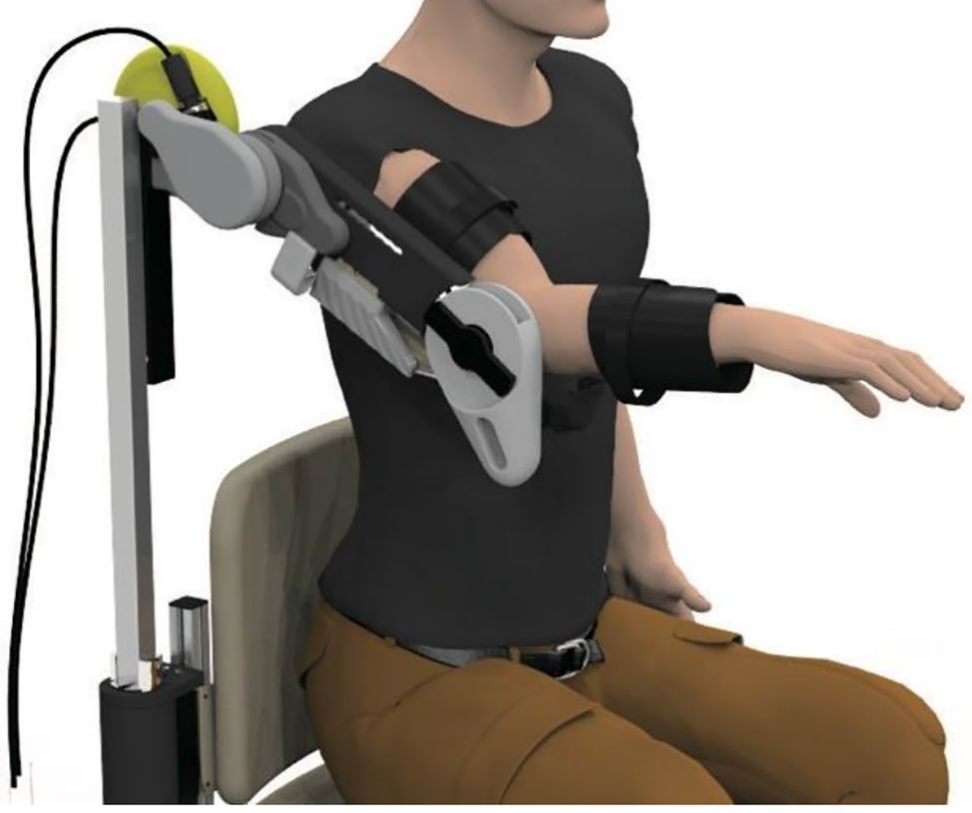
Shoulder elevation-assisted exoskeleton robot

This study used a shoulder elevation-assisted exoskeleton robot developed by Noda et al. [26] at the *Advanced Telecommunications Research Institute International* to perform the intervention (Fig. 1). This robot can assist in smooth shoulder elevation movements by supporting the elbow and forearm with belts and driving artificial muscles with pneumatic pressure. In this study, the robotic assist rate was adjusted from 0 (no assistance) to 100 (full assistance) based on the weight of the participant’s upper limb. Additionally, we integrate a surface electromyography (EMG) recording device and an electrical stimulator into the robot’s real-time control system. Although Fig. 1 shows only the right exoskeleton, the robot was equipped with both exoskeleton arms to provide intervention for right and left paralysis.

#### Optimal assist rate calculation model

The optimal robot assist rate was derived by identifying the change points using the co-contraction index (CCI) across the assist level, which is the arm’s gravity compensation level. It was calculated from the EMG data obtained from the anterior deltoid and biceps brachii muscles during shoulder elevation at baseline. The CCI is an indicator of abnormal movement. Generally, the CCI is calculated with pairs of opposing muscles (such as the biceps and triceps brachii). In contrast, it can also be calculated for pairs of abnormal coactivation muscles, such as the anterior deltoid and biceps brachii [27]. Furthermore, it has been reported that the CCI of the anterior deltoid and biceps brachii during shoulder flexion is highly correlated with spasticity in elbow flexion [28]. Therefore, we developed a method to derive the optimal robotic assist rate from the CCI of the anterior deltoid and biceps brachii muscles during shoulder flexion in patients with stroke.

As a preliminary preparation, skin treatment was performed (SkinPure, Nihon Kohden), which was applied to the anterior deltoid, middle deltoid, biceps brachii, and upper trapezius muscles. The hemiplegic upper limb was attached to the robot, and the weight of the hemiplegic upper limb was measured. We then performed the measurement task (five trials of shoulder elevation up to 90° of shoulder flexion) with a 100% assist rate (full assist) based on the weight of the hemiplegic upper limb. EMG was obtained during the measurement task. When the measurement task was performed, the assistance rate was reduced by 10%, and the EMG during the measurement task was repeated for up to 11 sets. The acquired EMG data were used to derive the optimal assist rate using the following procedure:

## Preprocessing

### Acquisition of muscle activity time-series data $E\left(t\right)$

Rectification was performed after subtracting the average from the EMG data.

- Filtering (10 Hz low-pass with an 8th-order Butterworth filter).
- Subtract the minimum value for baseline removal.

### Interpolation of angle data $A\left(t\right)$ to match sampling rate with EMG

Time-series flexion angle data were smoothed using 11-point moving averages.

- The flexion angle data were linearly interpolated in 0.001-second increments, which matched the EMG data.

### Elimination of noise caused by initial robot motion

- Eliminate $E\left(t\right)$ between 0.2 and 0.4 s after the initial robot motion.

## Visualization of EMG corresponding to angle data

- After removing duplicates, we defined ${t}_{max}$ as the moment when the arm angle reached its maximum.

An A-E curve was obtained by extracting $E\left(t\right)$ concerning $A\left(t\right|t<{t}_{max})$. In the case where duplicate data existed in $A\left(t\right|t<{t}_{max})$ (e.g., when arm elevation stopped for a moment), the latter was removed.

- Linear interpolation of A-E data in 0.1-degree increments.

## Derivation of the optimal assist rate

### Calculation of CCI

- We defined ${t}_{50}$ as the moment when shoulder flexion reached 50 degrees, and ${t}_{max}$ as the moment when $A\left(t\right)$ reached its degree maximum.

Calculate CCI from muscle activity $E\left(t\right)$ in the anterior deltoid and biceps brachii muscles as follows:

$$N(t) = \frac{{E(t)}}{{{E_{\max }}}}$$

$$C(t) = N(t|{t_{50}} < t \le {t_{\max }}) - \frac{1}{{{T_r}}}\int_{{T_r}} {N(t)dt}$$

$$CCI(t) = \min \left( {{C_{A.D.}}(t),{C_{B.B.}}(t)} \right)$$

Where ${E}_{max}$ represents the maximum muscle activity, defined as median value of $\text{m}\text{a}\text{x}\left(E\right(t\left)\right)$ at each trial, ${T}_{r}$ is the length of the rest duration, $C\left(t\right)$ is contraction rate in the specific muscle, and ${C}_{A.D.}$and ${C}_{B.B}$ represent the contraction rates obtained in the anterior deltoid and biceps brachii, respectively.

The time-averaged CCI was used as the representative value for each trial rate. Five representative CCIs are obtained for each assistive rate.

### Derivation of the optimal assist rate (Fig. 2)

**Fig. 2:**
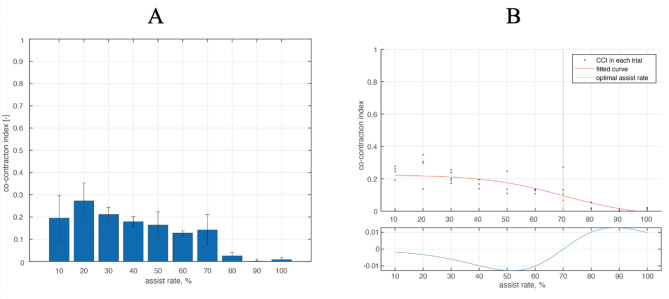
Derivation of the optimal assist rate

The calculated CCI values were fitted using an adjusted sigmoid function as follows:

$$f\left(r\right)=\frac{a}{1+{e}^{-b\left(r-c\right)}}+d$$

where *r* is the assistance rate, *a* the plateau of the curve (convergence value), *b* the gain of the sigmoid function, *c* the inflection point, *d* the CCI offset. The function is based on a sigmoid function and is adjusted such that the inflection point is outside the origin and the maximum value is scalable. Fitting was performed using nonlinear least-squares regression (Fig. 2-A).

The optimal assist rate was calculated as *r*, where the second derivative of the fitted curve was zero (Fig. 2-B).

#### Intervention

**Fig. 3:**
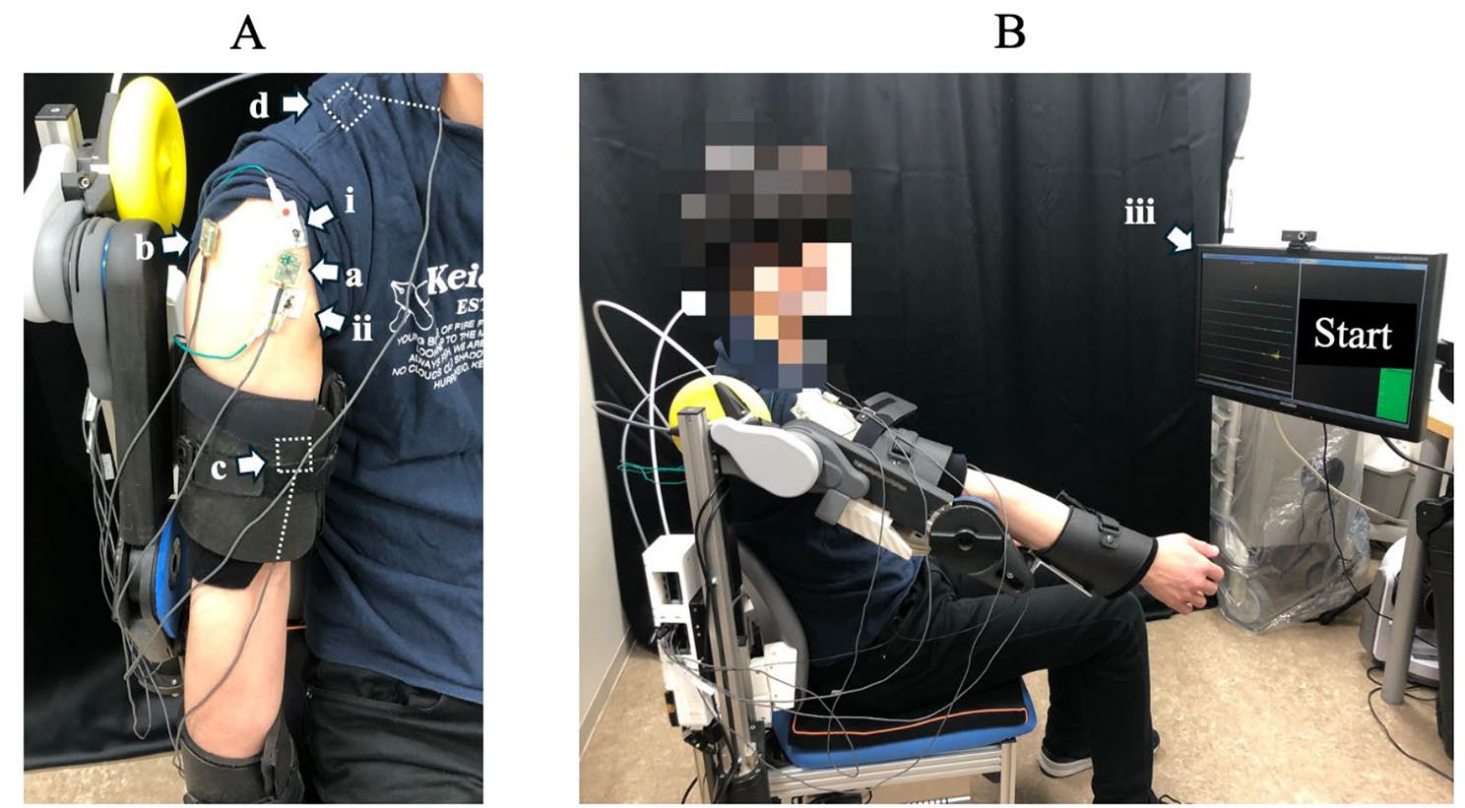
Experimental set-up: (**a**) EMG electrode of the anterior deltoid, (**b**) EMG electrode of the middle deltoid, (**c**) EMG electrode of the biceps brachii, (**d**) EMG electrode of the upper trapezius, (**i**) and (**ii**) electrical stimulation electrodes of the anterior deltoid, (iii) left, EMG waveforms; right, motions instructions

The intervention consisted of 100 robot-assisted shoulder elevation exercises (10 sets of 10 repetitions, approximately 40 min each) with a derived assist rate. Preparation for the intervention was similar to the pre-evaluation, with EMG electrodes attached to the anterior deltoid, middle deltoid, biceps brachii, and upper trapezius muscles, and the hemiplegic upper limb attached to the robot (Fig. 3-A). In addition to robotic assistance, muscle stimulation of the anterior deltoid was performed using an electrical stimulator at the start of shoulder elevation to achieve the synergy required for isolated shoulder elevation movement (Fig. 3-A). The pulse width, stimulation duration, and intensity of the electrical stimulation were set at 1 ms and 10 ms. The peak voltage of the stimulation pulse was adjusted to the minimum amplitude required to elicit muscle contraction. The participants performed repetitive shoulder flexion movements while viewing the EMG waveforms (Fig. 3-B).

### Assessment Protocol

All participants underwent clinical and kinematic assessments on the first and last days to investigate the effectiveness and safety of robotic therapy.

#### Effectiveness

##### Upper-extremity function

The upper-extremity function was assessed using the Fugl–Meyer assessment-upper extremity (FMA-UE) motor score. The FMA-UE consists of 30 motor function items and 3 reflex function items. The FMA-UE is scored on a 3-point ordinal scale (0 = cannot perform, 1 = partially performs, and 2 = completely performs), with higher scores indicating better motor function (total score: 0–66 points). The FMA-UE can be divided into four categories: A, shoulder/elbow/forearm (0–36); B, wrist (0–10); C, hand (0–14); and D, coordination/speed (0–6). The validity and reliability of the FMA-UE have been demonstrated previously [29].

##### Kinematic analysis

Azure Kinect DK (Microsoft) and dedicated software (ICpro-K2; Hu-tech C0, Ltd., Tokyo, Japan) were used to analyze the hemiplegic shoulder elevation. The participants performed maximum shoulder flexion tasks while keeping the elbow extended as much as possible while sitting and were recorded using Kinect. The data were preprocessed by spline interpolating lost data points and smoothed using a second-order Butterworth filter with a cut-off frequency of 5 Hz. The analysis sections were extracted as three-dimensional coordinate data using dedicated software. The maximum angle of voluntary shoulder flexion and elbow flexion ratio during shoulder elevation were analyzed from the extracted three-dimensional coordinate data. The maximum angle of voluntary shoulder flexion was calculated as the angle between a line perpendicular to the floor through the shoulder coordinates and a straight line from the shoulder to the elbow coordinates. The elbow flexion ratio during shoulder elevation was derived from the path length ratios of the three-dimensional coordinates of the shoulder (S), elbow (E), and hand (H), as follows:

$$\frac{\underset{SH}{\to }}{(\underset{SE}{\to }+ \underset{EH}{\to })} \times 100$$

This index decreases with increasing elbow flexion during shoulder elevation because of the proximity of the shoulder and hand (the maximum value is 100). The validity of calculating the shoulder flexion angle and path length ratio using Kinect has been established [30].

#### Safety

##### Adverse events

Medical interviews were conducted before and after the intervention to assess shoulder pain, additional physical impairment, and serious adverse events.

##### Shoulder pain

Shoulder pain was assessed using the pain-free passive shoulder flexion ROM and the visual analogue scale (VAS) for pain severity during voluntary or passive shoulder flexion. The pain-free passive shoulder flexion ROM was assessed by measuring the pain-free passive ROM of the hemiplegic shoulder flexion using a clinical goniometer, where a loss of range indicated increased pain [31].

### Statistical analysis

Before-and-after comparisons were conducted using the Wilcoxon signed-rank sum test for several parameters to examine the effectiveness and safety of robotic therapy. These parameters included FMA-UE, maximum angle of voluntary shoulder flexion, elbow flexion ratio during shoulder elevation, pain-free passive shoulder flexion ROM, and VAS score of voluntary/passive shoulder flexion. The FMA-UE A score was used to assess shoulder function. Regarding the elbow flexion ratio during shoulder elevation, the path length ratio was calculated by matching the maximum shoulder flexion angle to the lower pre- or post-intervention area, and the areas under the curve (AUC) were compared. All statistical analyses were performed using IBM SPSS Statistics (version 28.0; IBM, Tokyo, Japan). Statistical significance was set at *P* ≤ 0.05.

## Results

A total of 12 participants were recruited between October 2021 and October 2022. All 12 patients completed the procedure, including assessments before and after the intervention. Two participants were excluded from the analysis because the EMG of the biceps brachii was not obtained, and the robotic assist rate was determined from the CCI of the anterior deltoid and upper trapezius muscles.

### General characteristics of the participants

The general characteristics of the ten stroke survivors (five men and five women) who fulfilled the inclusion criteria are summarized in Table 1. The mean (standard deviation [SD]) age and the duration from stroke onset were 57.0 (5.5) years and 4.5 (3.1) years, respectively. The type of stroke was hemorrhage in nine patients, infarction in one, right paralysis in six, and left paralysis in four. The mean (SD) FMA-UE total and A scores pre-intervention were 18.7 (10.5) points and 14.8 (5.2) points, respectively.

**Table 1:** General characteristics and baseline upper-extremity function of the participants

Characteristics	Values
Number	10
Sex (men/women)	5/5
Age (years)	57.0 (5.5)
Duration from stroke onset (years)	4.5 (3.1)
Stroke type (hemorrhage/infarction)	9/1
Paralysis side (right/left)	6/4
FMA-UE Total score (0–66)	18.7 (10.5)
A score (0–36)	14.8 (5.2)
B score (0–10)	1.1 (2.3)
C score (0–14)	2.8 (3.8)
D score (0–6)	0.0 (0.0)

Values are presented as numbers or mean (standard deviation)

FMA-UE, Fugl–Meyer assessment-upper extremity

### Effectiveness

Table 2 presents the changes in each outcome. The mean (SD) FMA-UE A score showed a significant improvement from 14.8 (5.2) to 15.8 (5.4) points (*P* = 0.047). Regarding kinematic outcomes, there were significant improvements in the maximum angle of voluntary shoulder flexion (73.7 [34.2] to 84.9 [39.0]; *P* = 0.047) and AUC of the elbow flexion ratio during shoulder elevation (83.3 [6.6] to 88.5 [5.0]; *P* = 0.005).

**Table 2:** Changes in clinical and kinematic outcome measures

No	Robotic assist rate (%)	Upper-extremity function			Kinematic outcomes						Shoulder pain							
		FMA-UE A score (points)			Maximum angle of voluntary shoulder flexion (°)			AUC of elbow flexion ratio during shoulder elevation (%)			Pain-free passive shoulder flexion ROM (°)			VAS of voluntary shoulder flexion			VAS of passive shoulder flexion	
		Pre	Post		Pre	Post		Pre	Post		Pre	Post		Pre	Post		Pre	Post
1	80	12	11		64.0	68.3		76.6	83.3		135	140		0	0		3	0
2	50	19	21		102.7	140.3		84.2	90.1		125	125		0	0		0	0
3	50	21	21		133.4	137.9		89.7	96.9		130	150		0	0		0	0
4	85	10	12		23.9	19.7		96.1	97.0		135	140		0	0		0	0
5	85	8	9		26.0	32.5		86.0	88.7		110	110		1	0		0	0
6	85	14	14		77.5	87.1		77.7	81.8		105	115		0	0		5	1
7	90	10	11		64.3	56.8		71.3	86.4		145	145		1	0		0	0
8	60	13	13		48.3	87.5		82.9	84.1		110	135		0	0		0	0
9	66	16	20		83.6	104.2		83.0	89.9		110	120		0	0		0	0
10	50	25	26		113.7	114.9		85.7	86.8		140	140		0	0		0	0
Mean	70.1	14.8	15.8*		73.7	84.9*		83.3	88.5*		124.5	132.0*		0.2	0.0		0.8	0.1
SD	15.8	5.2	5.4		34.2	39.0		6.6	5.0		13.9	12.9		0.4	0.0		1.7	0.3

*p-value of < 0.05, compared post with pre using Wilcoxon signed-rank test

Abbreviations: AUC, areas under the curve; FMA-UE, Fugl–Meyer assessment-upper extremity; ROM, range of motion; SD, standard deviation; VAS, visual analogue scale

### Safety

No adverse events were observed across all participants during the study period. For shoulder pain, the pain-free passive shoulder flexion ROM improved significantly from 124.5 (13.9) to 132.0 (12.9) (*P* = 0.027). The VAS scores showed an improving trend in voluntary shoulder flexion (0.2 [0.4] to 0.0 [0.0]; *P* = 0.157) and passive shoulder flexion (0.8 [1.7] to 0.1 [0.3]; *P* = 0.180). Moreover, all patients showed either unchanged or improved shoulder pain outcomes (Table 2).

Additional file 1: Pre- and post-intervention values, gains, and statistical group differences of each outcome measures by the severity of upper-extremity paralysis.

## Discussion

### Summary of results

This study revealed the feasibility of using a shoulder elevation-assisted exoskeletal robot with a derived assist rate by identifying the change points using the CCI during shoulder elevation in stroke survivors with hemiplegic upper extremities. All 10 participants completed the 10-day intervention without adverse events such as shoulder pain and showed improved upper-extremity function.

### Effectiveness: improvement of Upper-Extremity function

This study showed a significant improvement in the FMA-UE A score and maximum angle of voluntary shoulder flexion in patients with severe upper limb motor paralysis. The average pre-intervention FMA-UE total score was 18.7 points in this study. In the upper-extremity impairment severity classification by FMA-UE total score, scores ≤ 19 points are severe, and 20–47 are moderate [32]. Thus, our participants are in the severe upper limb paralysis cluster. Generally, patients with severe motor impairments show poor functional improvement [33]. Robotic therapy for severe upper limb paralysis has also demonstrated that no significant benefit has been demonstrated [34]. Most robotic therapies benefit patients with moderate-to-mild upper limb paralysis [35].

Nevertheless, this study showed that robotic therapy for patients with severe upper-extremity paralysis significantly improved FMA-UE A scores by an average of 1.0 points and the maximum angle of voluntary shoulder flexion by an average of 11.2°. Criteria such as the minimally clinically important difference in these outcomes in severe upper-extremity paralysis have not been established and are generally difficult to consider. Nevertheless, our results, which show a significant improvement in upper limb motor function in patients with severe upper-extremity paralysis, are novel and meaningful. Comparison between the severe and moderate upper extremity paralysis groups showed some improvement in the severe group (Additional file 1). This improvement may be attributed to the robotic assistant’s repetitive vertical shoulder movements (shoulder flexion). In previous studies, gravity- or upper-limb weight-supported robotic exercises for patients with stroke provided functional recovery of the upper extremities [36–39]. However, none of these are vertical movement supports. Therefore, the robotic therapy in this study is one of the few interventions that can provide repetitive movements to significantly improve motor function, even in cases of severe upper-extremity motor impairment.

### Effectiveness: improvement of abnormal Movement

Moreover, this study significantly improved the elbow flexion ratio during shoulder elevation. The AUC of the elbow flexion ratio during shoulder elevation improved by an average of 5.2% in all participants. In other words, all patients could perform shoulder flexion with elbow extension compared to the pre-intervention period. This result may be attributed to the robotic assist rate derived from the CCI between the anterior deltoid and the biceps brachii. Previous studies have reported that robotic therapy reduces the co-contraction between the anterior deltoid and biceps brachii. Previous studies have also reported that the CCI between the biceps and triceps brachii (agonist and antagonist muscles) is reduced by robotic therapy [40–42]. However, this is the first report demonstrating a reduction in abnormal movements, rather than a relationship between agonist and antagonist muscles, through kinematic assessment. Thus, robotic therapy with an optimal assist rate has shown effectiveness in improving upper-extremity function and reducing abnormal movements.

### Safety

No adverse events were associated with this intervention, and the pain-free passive shoulder flexion ROM significantly improved by a mean of 7.5°. The incidence of shoulder pain in hemiplegic shoulders has been reported to be 10–22% [43]. Moreover, shoulder pain has been reported to affect physical function adversely [43–46], activities of daily living [45, 47, 48], and quality of life [49]. Hence, it is essential to provide appropriate difficulty that does not cause pain when intervening in hemiplegic shoulders. The results showed that shoulder pain outcomes were maintained or improved in all participants; thus, the robot’s safety was demonstrated.

### Strength

The strength of this study is the apparent improvement in upper-extremity function and abnormal movements (elbow flexion ratio during shoulder elevation) without shoulder pain. This result indicated that the derived robotic assistance rate was appropriate. When the assistance rate is too high (excessive assistance), participants tend to exhibit “slacking,” characterized by a lack of voluntary movement and a lack of improvement in upper-extremity function [23, 24]. Conversely, a robotic assist rate that is too low may increase elbow flexion during shoulder elevation, worsen shoulder pain outcomes, and cause adverse events. However, neither of these events occurred, and both outcomes improved. The calculation method developed in this study can be used to derive an appropriate robot assist rate.

### Limitations

The limitations of this study include the small sample size and the absence of a control group, which makes it difficult to generalize the findings. Although this study showed improvements in upper-extremity function, it is unclear whether these results were because of robotic rehabilitation, as the patients also received conventional training. However, robotic therapy consists of interventions focusing on shoulder flexion, whereas conventional training is a traditional proximal and distal upper-extremity intervention. Therefore, the significant improvement shown by the FMA-UE A score indicates the usefulness of robotic therapy. Future studies are required to identify the effectiveness of robotic therapy in larger RCTs.

## Conclusion

A model was established to derive an appropriate assist rate calculation model by identifying the change points and using the CCI to derive the robotic assist rate. Furthermore, robotic rehabilitation using a shoulder elevation-assisted exoskeletal robot based on a derived optimal assist rate demonstrated the possibility of safely improving the motor function of hemiplegic upper limbs in patients with stroke.

### Electronic supplementary material

Below is the link to the electronic supplementary material.

**Additional file 1:** Pre- and post-intervention values, gains, and statistical group differences of each outcome measures by the severity of upper-extremity paralysis

## Data Availability

The datasets used and/or analysed during the current study are available from the corresponding author on reasonable request.

## Abbreviations

AUC: areas under the curve
CCI: co-contraction index
EMG: electromyogram
FMA-UE: Fugl–Meyer assessment-upper extremity
ROM: range of motion
SD: standard deviation
VAS: visual analogue scale
