# Supplementary material for: Optimizing shoulder elevation assist rate in exoskeletal rehabilitation based on muscular activity indices: a clinical feasibility study
Source: BMC Neurol. 2024 May 9;24:144. doi: 10.1186/s12883-024-03651-x (PMC11080160; doi:10.1186/s12883-024-03651-x)
Supplement: Supplementary file 1 — Additional file 1: Pre- and post-intervention values, gains, and statistical group differences of each outcome measures by the severity of upper-extremity paralysis [file 12883_2024_3651_MOESM1_ESM.docx]

**Additional file 1:**

To compare differences in severity of upper extremity paralysis, we classified FMA total scores of ≤19 into the severe group and those of 20–46 into the moderate group, using the criteria of Woodbury ML et al [1]. The Wilcoxon Signed Rank test was applied to examine pre- and post- intervention changes in each group, comparing mean baseline values with the values at the end of the ten days program. The differences of functional gains between both groups were analyzed with the Mann-Whitney U test. All statistical analyses were performed using IBM SPSS Statistics (version 28.0; IBM, Tokyo, Japan). Statistical significance was set at *P* ≤ 0.05.

Of the ten participants in the analysis, seven in the severe upper-extremity paralysis group and 3 in the moderate upper-extremity paralysis group. The severe group showed significant improvements in AUC of elbow flexion ratio during shoulder elevation (*P* = 0.018) and pain-free passive shoulder flexion ROM (*P* = 0.041) from pre- to post- intervention. In the moderate group, there were improvements in all outcome measures from pre- to post- intervention, but none were statistically significant. The pre FMA-UE A score and pre maximum angle of voluntary of shoulder flexion of the severe group were significantly lower than those of the moderate group (*P* < 0.05). However, gains from pre- to post- intervention were not significantly different between either group.

Reference

[1] Woodbury ML, Velozo CA, Richards LG, Duncan PW. Rasch analysis staging methodology to classify upper extremity movement impairment after stroke. Arch Phys Med Rehabil. 2013;94:1527–33. https://doi.org/10.1016/j.apmr.2013.03.007

Additional Table 1: Pre- and post-intervention values, gains, and statistical group differences of each outcome measures by the severity of upper-extremity paralysis

|  | Severe upper-extremity paralysis group (n = 7) | | | |  | Moderate upper-extremity paralysis group (n = 3) | | | |  | Group differences (*P*-value) | |
| --- | --- | --- | --- | --- | --- | --- | --- | --- | --- | --- | --- | --- |
| Outcome Measures | Pre | Post | Gain | *P*-value |  | Pre | Post | Gain | *P-*value |  | Pre | Gain |
| FMA-UE A score | 11.9 (2.5) | 12.9 (3.3) | 1.0 (1.5) | 0.131 |  | 21.7 (2.5) | 22.7 (2.4) | 1.0 (0.8) | 0.180 |  | 0.017^‡^ | 0.833 |
| Maximum angle of voluntary of shoulder flexion | 55.4 (21.9) | 65.2 (28.6) | 9.8 (14.7) | 0.128 |  | 116.6 (12.7) | 131.0 (11.4) | 14.4 (16.4) | 0.109 |  | 0.017^‡^ | 1.000 |
| AUC of elbow flexion ratio during shoulder elevation | 81.9 (7.3) | 87.3 (4.8) | 5.4 (4.6) | 0.018* |  | 86.5 (2.3) | 91.3 (4.2) | 4.7 (2.7) | 0.109 |  | 0.267 | 1.000 |
| Pain-free passive shoulder flexion ROM | 121.4 (15.1) | 129.3 (12.9) | 7.9 (8.0) | 0.041* |  | 131.7 (6.2) | 138.3 (10.3) | 6.7 (9.4) | 0.317 |  | 0.517 | 0.667 |
| VAS of voluntary shoulder flexion | 0.3 (0.5) | 0.0 (0.0) | -0.3 (0.5) | 0.157 |  | 0.0 (0.0) | 0.0 (0.0) | 0.0 (0.0) | - |  | 0.517 | 0.517 |
| VAS of passive shoulder flexion | 1.1 (1.9) | 0.1 (0.3) | -1.0 (1.6) | 0.180 |  | 0.0 (0.0) | 0.0 (0.0) | 0.0 (0.0) | - |  | 0.517 | 0.517 |
| *p value of < 0.05, compared post with pre using Wilcoxon signed–rank test.  ^‡^p value of < 0.05, compared between severe group and Moderate group using Mann-Whitney U test. | | | | | | | | | | | | |
| Abbreviations: AUC, area under curve; FMA-UE, Fugl-Meyer assessment-upper extremity; ROM, range of motion; VAS, visual analogue scale. | | | | | | | | | | | | |
|  | | | | | | | | | | | | |
